# An Innovative Method to Analyse the Geometrical Accuracy of Ti6Al4V Octet-Truss Lattice Structures

**DOI:** 10.3390/ma16062372

**Published:** 2023-03-16

**Authors:** Costanzo Bellini, Rosario Borrelli, Francesco Di Caprio, Vittorio Di Cocco, Stefania Franchitti, Francesco Iacoviello, Luca Sorrentino

**Affiliations:** 1 Department of Civil and Mechanical Engineering, University of Cassino and Southern Lazio, 03043 Cassino, FR, Italy; 2 CIRA—Italian Aerospace Research Centre, Via Maiorise, snc, 81043 Capua, CE, Italy

**Keywords:** electron beam melting, titanium, thin structure, geometrical accuracy

## Abstract

Metal lattice structures manufactured utilising additive techniques are attracting increasing attention thanks to the high structural efficiency they can offer. Although many studies exist on the characterisation of massive parts in Ti6Al4V processed by Electron Beam Melting (EBM), several investigations are necessary to characterise the Ti6Al4V lattice structures made by the EBM process. The objective of this paper is to develop a measurement method to assess the dimensional accuracy of Ti6Al4V octet truss lattice structures manufactured by EBM technology. Beam specimens with a 2 mm diameter and different growth orientations with respect to the build direction were analysed. The geometry differences between the designed and the manufactured beam specimens were highlighted. Two effects were identified: (i) The EBM-manufactured beams are generally thinner than the designed ones, and (ii) the shape of the section was found to be almost circular for the beam specimens oriented at 45° and 90°; on the contrary, the section of the horizontal beam (0°) cannot be considered circular.

## 1. Introduction

In the past 30 years, there has been an extraordinary development in additive manufacturing (AM) technologies, which has gradually moved from research in academia to production in industry, becoming standard practice in many industrial fields [1]. The American Society for Testing and Materials (ASTM) defines AM as “a process of joining materials to make objects from 3D model data, usually layer upon layer, as opposed to subtractive manufacturing methodologies” [2]. Many benefits can be obtained using additive technologies instead of traditional manufacturing processes, such as the possibility to design any complex shape, high process automation, lower energy, and environmental costs. The AM technologies allow designers to topologically optimize mechanical components for weight saving or to manufacture assemblies with a lower number of parts [3]. Another promising application of AM consists in the manufacturing of lattice structures or cellular materials with a prescribed microarchitecture. Lattice materials have attracted the research community for many years for their ability to provide exclusive and high mechanical properties [4,5,6,7] and to design their structure in order to fulfil specific and multifunctional design constraints [8,9]. Their architecture is formed by an array of spatial periodic unit cells. the mechanical and physical behaviour of the lattice structure may be modified by changing the dimensions of the beams and the unit cell topology [10]. Heat exchangers, energy absorbers and lightweight structural components are lattice structures’ potential applications [11,12,13].

Despite the promising capabilities of lattice structures, their full exploitation has been limited by the lack of adequate manufacturing techniques that are able to produce, at adequate costs, complex lattice networks. The manufacturing of a lattice structure through traditional techniques is quite expensive because several cutting and welding phases are required [14]. Thanks to the maturation of AM techniques in recent times, the interest in lattice structures is increased and numerous studies have been performed to understand and characterise the behaviour of such structures manufactured by additive technologies [15,16,17,18].

Nevertheless, although additive manufacturing has enabled the production of metallic lattice structures with tailored mechanical properties, several problems related to the AM processes still exist, affecting the quality of the struts. Metal AM techniques can be divided into powder bed fusion (PBF) techniques and directed energy deposition (DED) techniques [1,19]. The AM processes based on the PBF technique, thanks to the ability to build workpieces with complex details and assure good dimensional control, are the most mature and widely used metal additive manufacturing processes. PBF uses a high-energy power source to selectively melt or sinter a metallic powder bed. Depending on the type of power source, PBF can be further divided into two major techniques: selective laser melting (SLM) [20], which uses a high-intensity laser, and electron beam melting (EBM) [21], which uses an electron beam. Both processes need a building platform to hold the powder.

Regarding the EBM process, several studies [22,23,24,25,26] have investigated the mechanical characterisation of the Ti6Al4V alloy, one of the most suitable materials to be processed by EBM. In general, Ti6Al4V parts made in EBM have very fine microstructures and a porosity lower than 0.2%, which can be furtherly reduced by Hot Isostatic Pressure (HIP) treatment. If compared to wrought counterparts, EBM parts typically have equivalent or superior mechanical properties: high yield stress (YS), ultimate tensile stress (UTS), and higher hardness but a lower ductility [27]. As a main drawback, the EBM produces parts with a high roughness [28,29]. The dimensional accuracy of the EBM process and its repeatability have been studied on Ti6Al4V samples in [30]. Measurements performed on different builds, made in the same conditions, were found to be very similar, demonstrating very high repeatability of the EBM process in terms of dimensional accuracy. Moreover, the investigation showed that the orientation of the samples and their location in the build chamber are significant parameters affecting the part dimensional accuracy.

All the above-mentioned studies on the characterisation of Ti6Al4V processed by EBM have focused their attention on the use of the process for massive components that have diameters greater than 2 mm. On the other hand, several investigations have been carried out to characterise the behaviour of Ti6Al4V lattice structures made by the EBM process [31,32,33,34]. In this case, a different melting strategy (net theme) is used in the process with respect to the strategy used in massive components (melt theme). As confirmed by literature studies, the EBM process may be advantageously employed to manufacture lattice structures in Ti6Al4V, but some manufacturing imperfections, such as deviation from the nominal geometry and internal porosity, need to be further investigated in order to improve the quality of the structure and to ensure repeatability and reproducibility of the process which are crucial especially for critical safety applications. Indeed, the in-service performance implications of undersized beams in fully stressed lattice structures are potentially very serious. A decrease in the cross-sectional area will result in a lower axial load capacity of the beam which compromises the performance of an entire structure [35]. Thus, developing methods to analyse the geometrical accuracy of additively manufactured lattice structures has become an active area of research. The objective of this paper is to develop a measurement method to assess the geometrical accuracy of Ti6Al4V octet truss lattice structures manufactured by EBM technology. The developed method was validated against tomography results and then used to investigate the typical manufacturing imperfections as a function of the truss orientation. Several samples have been designed and manufactured by EBM at different orientations with respect to the start plate plane, in order to emulate the trusses of a lattice structure. The final aim is to provide guidelines for designers in order to consider the effects of geometrical imperfections during the design process of a lattice structure.

## 2. Materials and Methods

The characteristics of the material used in this study, the process parameters, the test specimens and the method used for assessing dimensional accuracy will be given in the next paragraphs.

Specimens were manufactured by using Ti6Al4V gas atomised powder with spherical morphology. The spherical shape contributes to improving flowability and thus ensures high build rates and part accuracy [36]. Powder characterisation was performed and the results are reported in a previous study [37]. The specimens used in the present study were designed in order to show the same geometry of the beam composing an octet-truss structure. The specific elementary unit is composed of a circular section having a diameter d equal to 2.0 mm. The length of the calibrated section is not particularly important for this study: it must be sufficiently representative for the analysis, and for this reason, a length of 25 mm was chosen.

**Figure 1:**
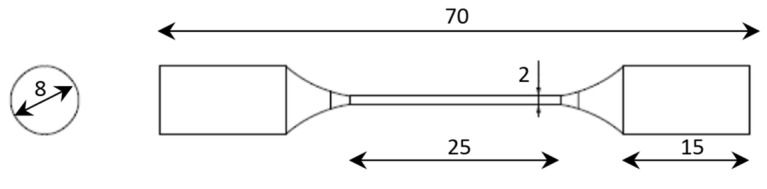
Beam specimen geometry (dimensions in mm).

The specimen geometry is shown in Figure 1. The software Solid Edge was used to generate the Computer Aided Design (CAD) model of the beam specimen. The CAD model was provided in two separate files representing, respectively, the specimen heads and the gauge length, in order to assign different process themes to the different parts as will be described afterwards.

**Figure 2:**
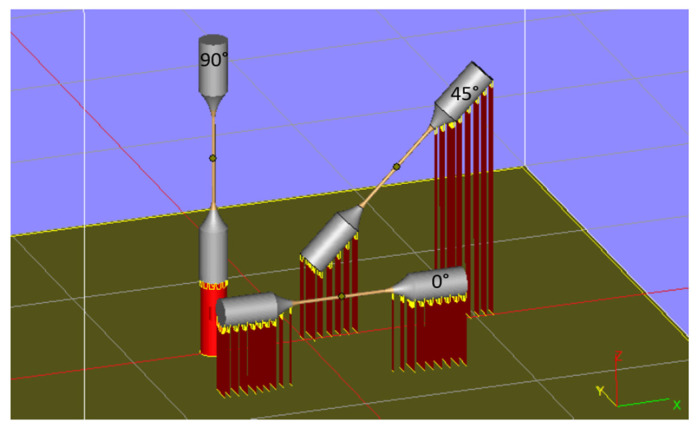
Growth orientation investigated in Magics Materialise environment.

The specimen CAD model was transferred to Magics Materialise software for placement on the building platform. In order to evaluate the influence on the dimensional accuracy of the specimens build direction, n. 6 specimens for each of the chosen growth directions were printed. As shown in Figure 2, three different directions of building with respect to the plane of the start plate (XY) were chosen: 0°, 90° and 45°. The 0° oriented samples were built horizontally, and the 90° oriented samples were built vertically (Figure 2). These orientations were chosen in this way because they are representative of those ones found in a lattice structure. In total, n. 18 specimens were manufactured.

Also, Magics Materialise software was used for wafer supports generation. Support structures, required in the EBM technology to help the heat energy dissipation and to decrease the geometric imperfections such as curling or warping, were created only for the specimen heads. The supports were 30 mm in length and had a line distance of 3 mm, as shown in Figure 2.

All the specimens were scaled according to the ARCAM recommended scale factors (1.0092 for x and y directions and 1.0132 for z direction) in order to take into account the thermal shrinkage occurring after the melting.

Afterwards, the STL files were sliced by using the software “Materialise Build Processor” and choosing a layer thickness of 50 μm. The output of the slicing process is the ARCAM Build Processor (ABP) file which is an archive of all the data required to run a build in an ARCAM machine.

The process themes were set through the “EBM control 3.2” software installed on the EBM system. The “automatic” operating mode of the EBM system was selected, and the following n.4 standard process themes for Ti6Al4V alloy and layer thicknesses of 50 μm were used:

1. “Ti6Al4V-PreHeat-50μm”: to control the phase of preheating the whole powder bed.
2. “Ti6Al4V-Melt-50μm”: to melt the solid part of the specimen (heads).
3. “Ti6Al4V-Net-50μm”: to melt the lattice part of the specimen (gauge length).
4. “Ti6Al4V-Wafer-50μm”: to manufacture the supports.

These process themes vary electron beam parameters during the build following an algorithm optimised by the manufacturer with the final aim to obtain parts with consistent properties. Since the algorithm is covered by copyright, beam current and beam speed time-dependent diagrams are hidden from users. A line offset of 0.1 mm was set.

The Electron Beam Melting system used in this study was the Arcam A2X, available at the Italian Aerospace Research Centre (CIRA). The Arcam A2X is designed to process titanium alloys as well as materials that require elevated process temperatures, e.g., titanium aluminide and Alloy 718, which makes it suited for both production and materials R&D. This EBM platform offers a build envelope of 210 × 210 × 380 mm. The build chamber of the Arcam A2X is specifically designed to withstand extremely high process temperatures, up to 1100 °C. In Arcam A2X, the EBM process was carried out in vacuum (5 × 10^−6^ mbar in the column).

**Figure 3:**
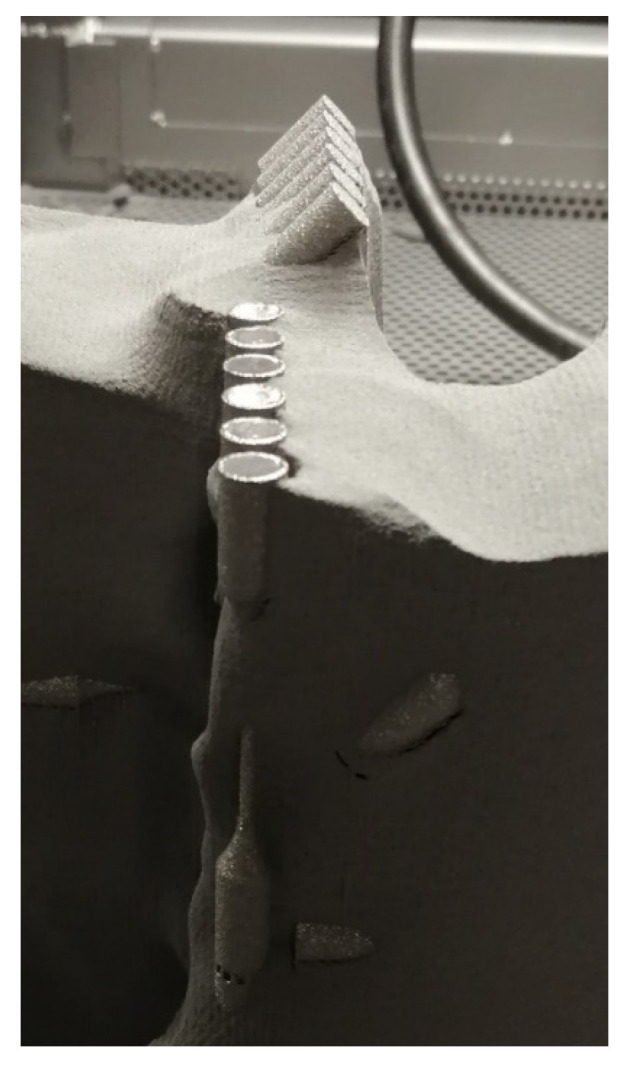
Post-running operation—depowdering of beam specimens in PRS.

**Figure 4:**
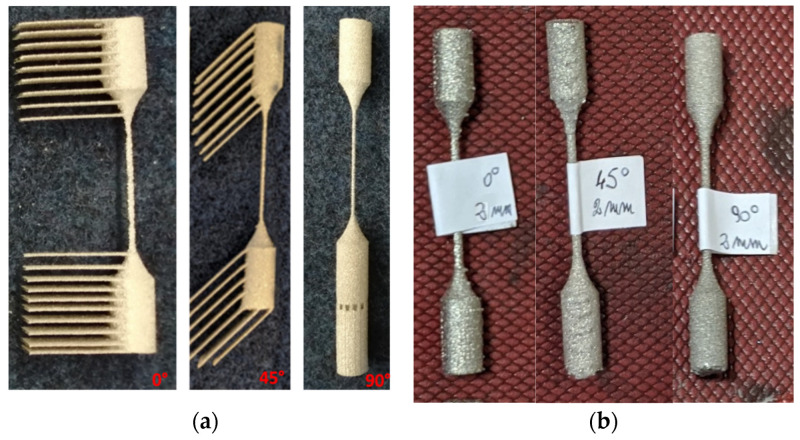
(**a**) Beam specimens before removing supports. (**b**) Beam specimens after removing supports.

After the job was completed, the build was moved with a trolley directly to the powder recovery system (PRS) to remove and recover the sintered powder from around the components (Figure 3) which was then sieved and saved for future use. After cleaning in the PRS was completed (Figure 4a), the wafer supports were removed as showed in Figure 4b.

**Figure 5:**
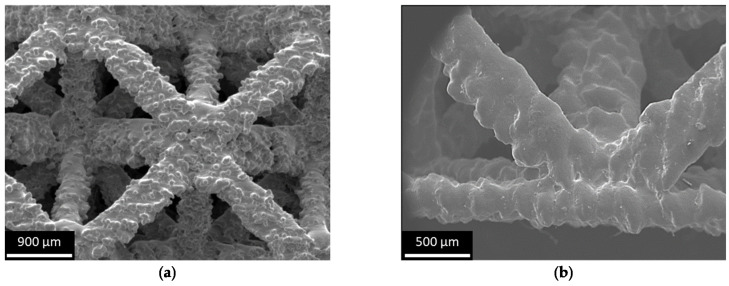
SEM micrography of the produced structures: (**a**) a node connecting some beams; (**b**) connection between skin and beams.

Figure 5 describes the evaluation of produced specimens using micrographs taken by a Philips 505 SEM. In Figure 5a, it can be noted that the surface of the beams was found to be irregular, and the diameter of the different beams varied depending on their orientation. This mismatch was attributed to processing phenomena, as explained in the Results section. In Figure 5b, it can be noted that the thickness of the skin was uniform, and the skin itself was planar. The interpenetration of the core into the skin was good, with the beams parallel to the skin partially enclosed in the skin.

**Figure 6:**
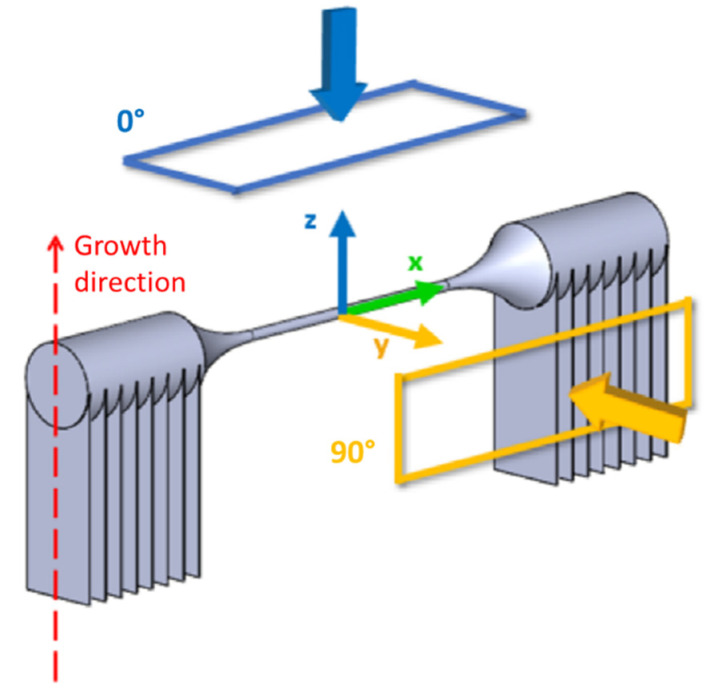
Reference direction for image acquisition.

After the manufacturing of the specimens, their dimensions were evaluated by using an image processing technique. The images of the specimens were taken using a Nikon SMZ800 stereomicroscope with 10× magnification. Two photographs were taken for each specimen: one along the direction parallel to the growth direction, that is, towards the normal to the start plate, named 0° direction, and another perpendicular to the former one, called 90° direction. The reference directions for image acquisition are represented in Figure 6 for the horizontal specimen, the 0° specimen.

**Figure 7:**
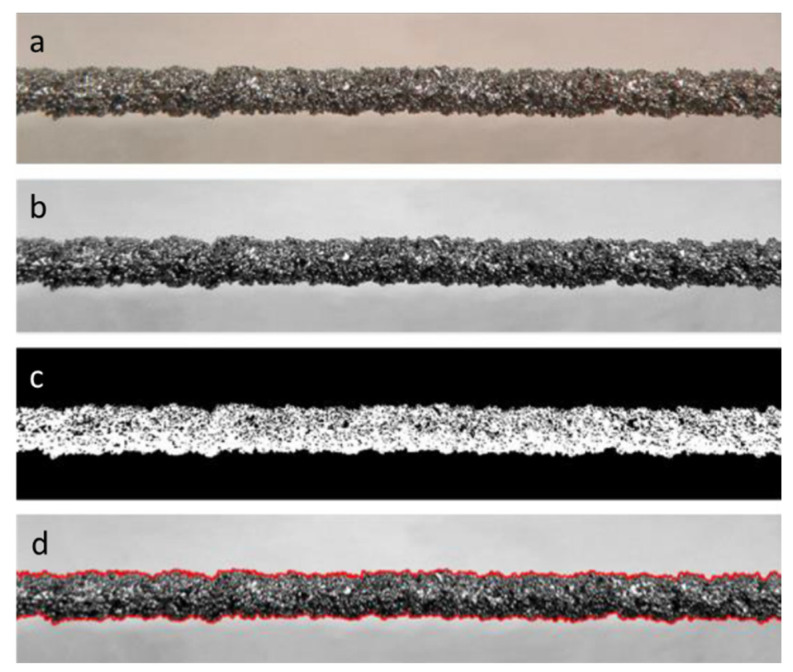
Image elaboration sequence: (**a**) cutting of unused areas; (**b**) conversion to grayscale; (**c**) binarization; (**d**) profile identification.

Then, the image was elaborated by using the Matlab Image Toolbox, according to the following procedure: firstly, unused areas were cut from the image for working only on the specimen area, then the image was converted to greyscale and then binarized. This last step is necessary to clearly distinguish in the image the object of interest from the background. Finally, the coordinates of the points representing the boundary of the specimen were identified and exported. The elaboration sequence is presented in Figure 7.

In order to verify the reliability of the proposed measurement method, a comparison with the measurements obtained from a X-ray Computed Tomography (XCT) analysis on the specimens is presented. The XCT analysis is one of the so-called Non-Destructive Testing (NDT) technologies, i.e., all the methods and techniques for guaranteeing and inspecting the quality of the product without altering the material. The XCT technique allows viewing sections of the body under examination in planes parallel to the direction of propagation of the radiation. By composing the tomographic images taken on different planes, placed at predefined distances, it is possible to reconstruct a three-dimensional image [38]. X-ray Computed Tomography (XCT) has become a common tool for dimensional analysis as it allows for non-destructive internal and external measurements [39].

The beam specimens were analysed by X-ray tomography using a 300 kV GE model V|TOMEX|M tomograph micro-focus tube suitable to scan highly absorbing materials. Data were acquired and elaborated by Phoenix Datos| x 2.0 software, and the reconstructed 3D volume was exported in 32-bit files and visualised by Volume Graphics Studio MAX 2022.3 software. In order to obtain the samples’ tomography images resolution with the voxel size of 44.68 μm, the source power of the machine was set to 85 W.

In order to fully characterise the outer structure of the manufactured trusses, the beam specimens were scanned at every 0.12° of the circumference; hence, 3000 profiles were analysed for each sample. For each beam specimen, the surface of the gauge length was approximated by a cylinder obtained by using the gaussian least squares method. For each cylinder, the average diameter and the coefficient of variation were measured.

## 3. Results

The measurement methodology exposed in paragraph 1.5 lead to the results reported in Table 1, where the average diameter for each orientation was computed as the mean value between the average diameter of the two acquisitions (0° and 90° measuring direction). First of all, it can be noted that the coefficient of variation was equal to about 3% for the specimens with a growth direction of 45° and 90°, but it was more elevated for the 0° specimens. This result demonstrates a good uniformity of the diameter along the entire length of the specimens for the former ones, while special considerations will be highlighted for the latter ones later in the article. Moreover, it must be underscored that the average diameter is thinner than the nominal one, and the deviation varied from more than 2.5% for the specimen with an orientation of 0°, to more than 15.5%, for the specimen with an orientation of 90°. One of the reasons why the beam diameter is thinner than the nominal one is due to the lack of the contouring phase in the net process theme, resulting in a more irregular cross-section with the respect to that one obtained by the melt process theme. Moreover, the scale factors used in this study are recommended by ARCAM for parts processed with the melt process theme, but there are no indications in the literature regarding their effectiveness on lattice structures processed with the net process theme. This geometrical reduction must be taken into consideration for designing purposes [40,41,42]. In the same table, the average diameter and the coefficient of variation values measured through X-ray tomography are reported and compared with the results obtained by the proposed methodology. Also, in this case, the specimens resulted thinner than wanted, being the deviation from the nominal diameter between 10% and 15%. Moreover, it can be noted that the percentage difference between the values obtained through the different methods is very slight, so the proposed methodology can be deemed reliable. In fact, in the case of the specimens with an orientation of 90°, the difference is negligible, while for the 0° specimens the difference is quite elevated, but no more than 10%.

However, special consideration must be emphasised for the specimen with an orientation of 0°. To this end, in Table 2, results obtained through the method proposed in this work are presented for both measurement directions. For the 0° oriented specimen, the diameter evaluated from the direction perpendicular to the start plate (0°) was thinner than expected, with a reduction of 12.85%, but the other measurement direction (90°), that is perpendicular to the growth direction, showed a thicker beam, with an increase of 7.69% with respect to the nominal geometry. Moreover, the coefficient of variation in the last case is more than 4%, which is the highest value found in this work. The aspect ratio of the specimen cross-section, that is the ratio between the average diameter evaluated in the 90° measurement direction and the other direction, is equal to almost 1 for the 45° and 90° specimens, while it was higher for the 0° specimens, denoting the lack of circularity for the latter ones. The reasons why the section of the horizontal beam (0°) cannot be considered circular are:

- In the specimen oriented at 90° the cross-section is influenced exclusively by the scale factors Fx and Fy which are equal to each other, while in the specimen oriented at 0° the cross-section is also influenced by the scale factor Fz which is greater than the other two;
- The shape of the cross-section is affected by the thermal history during the EBM process. For the specimen oriented at 90° and at 45° the thermal flux goes mainly through the beam length resulting in a relatively homogeneous roughness. For a beam oriented at 0°, the thermal flux is dissipated through the sintered powder, leading to an over-melting on the down-facing side and an even higher roughness according to [43].

This fact may explain the discrepancy found in the values determined by the different measuring methods for the 0° specimens.

**Figure 8:**
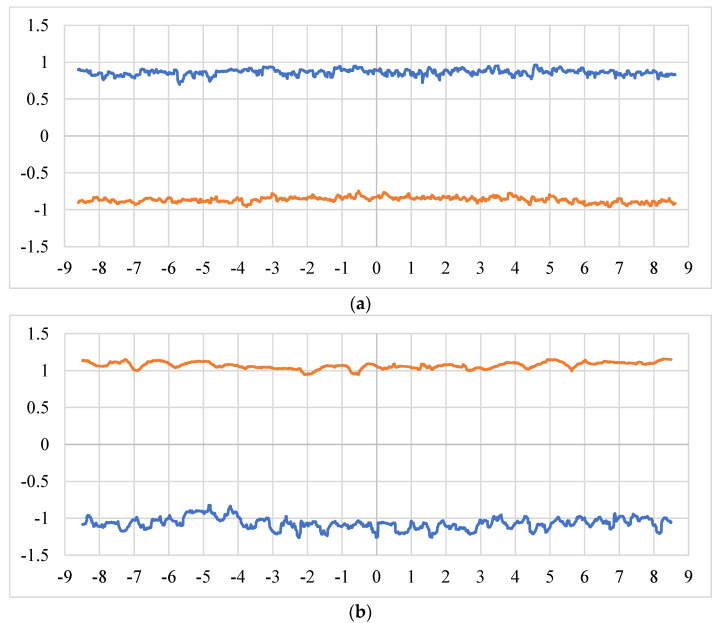
Profiles of the specimen with an orientation of 0°: (**a**) 0° measurement direction; (**b**) 90° measurement direction.

To deeply analyse this phenomenon, both the 0° and 90° profiles corresponding to the 0° oriented specimen are reported in Figure 8a,b, respectively. It is precisely in the latter that a clear difference is evident between the geometry of the upper and lower border: the former appears visibly more linear than the latter. There are studies in the literature in which the phenomenon is attributed to the problem of over-melting, which is frequent for horizontally printed specimens [43,44]. Further visual analysis of the two images confirms that the equiaxiality of the cross-section was not guaranteed. Therefore, the 0° oriented specimen showed a cross-section that is stretched downwards, in the opposite direction to that of growth. This last phenomenon is confirmed by the high roughness characterising the lower profile. Moreover, Figure 8a highlights the reduction of the specimen cross-section, as explained before, and an elevated roughness, that may be responsible for a reduction of the mechanical characteristics of the printed material, especially for the fatigue performances.

**Figure 9:**
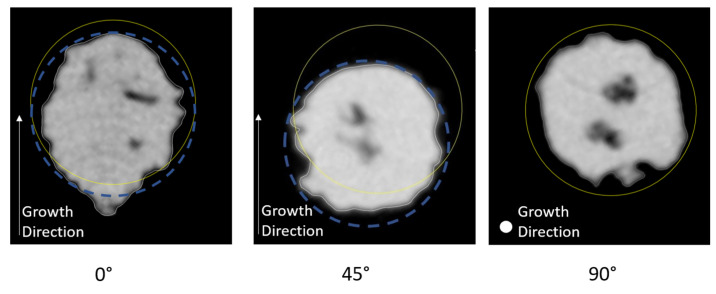
X-ray images of cross-section variation with orientation and comparison with the nominal cross-section.

The validity of the results obtained with the proposed measuring method was confirmed by the results obtained by tomographic investigations. Indeed, the shape of the cross-section for horizontally built specimens differs significantly from that of one of the specimens built with an orientation of 45° and 90°, as shown in the X-ray images of Figure 9. In such a figure, the specimens cross-sections at the centre of the gauge length are shown, nevertheless, the cross-sections of the beam specimens are almost the same along the whole specimens gauge length for each orientation investigated. The yellow outline is the nominal section of the specimen (diameter 2 mm). The blue dotted line for the 45° oriented specimen represents the CAD model nominal cross-section translated to circumscribe the tomographic image, according to the method described in Section 2. The following considerations can be drawn:

- A rather equiaxed cross-section was found for vertical and 45° oriented specimens, whereas for horizontally built specimens, the cross-section was found elongated along the build direction. It can be seen that for the specimens with an orientation of 0°, the real cross-section comes out from the nominal section in the direction opposed to the build direction.
- The 90° oriented specimen is well-centered with respect to the corresponding CAD model; conversely, the 45° oriented specimen presents a strong misalignment with respect to the nominal cross-section. Nevertheless, for the 45° oriented specimen, although the cross-section is translated from the nominal position, its shape is rather circular.
- The specimens are smaller than desired, with the exception of the horizontal specimens that only in the direction parallel to the growth direction are larger than desired.

## 4. Conclusions

In the present work, the geometrical characterisation of some beam specimens, representing the elements of a lattice structure, has been carried out. In fact, in previous studies, it has been demonstrated that there is a dimensional deviation between the nominal and the produced structures, and it is responsible for the decrement of the expected mechanical properties.

A new methodology based on image processing was developed to evaluate the diameter of the produced cylindrical beams. In particular, this methodology consisted in taking micrographs of the specimen, orthogonal to its axis, and then extracting the profiles of the part. Finally, by measuring the distance between two homologous points, belonging to the upper and lower profiles, it was possible to evaluate the diameter relevant to each section. The main advantage of the proposed method, based on image processing, consists in being less expensive compared to those usually employed in industrial applications, such as X-ray Computer Tomography, despite being able to maintain the same accuracy and effectiveness.

The results clearly highlighted the presence of irregular profiles and their constant undersizing compared to the nominal geometry. In fact, reductions in diameter with respect to the nominal geometry of around 15% were observed. This is very detrimental, as this deviation is amplified when considering tensile or flexural strength since, in this case, the second or fourth power of the diameter must be considered, respectively. Moreover, for horizontally built specimens, a certain difference between the diameter evaluated from the growth direction and the diameter measured from the orthogonal direction was found, being the former larger than the latter. The results show that the scaling factors used in the present study, and recommended by ARCAM for massive parts, are not effective for lattice structures. Consequently, further developments are necessary to optimize the scale factors in order to improve the dimensional accuracy of lattice structures manufactured by EBM.

Finally, the profiles of the specimens revealed an accentuated roughness, which in some cases generated real notches. These, as is well known, can reduce the mechanical characteristics of the printed part, especially its fatigue strength.

Results obtained by the proposed method were confirmed and supported by tomographic analysis of the beam specimens.

## Figures and Tables

**Table 1:** Average diameter for the produced specimens.

Growth Direction [°]	Proposed Method			X-ray Tomography			X-ray Tomography/Proposed Method Difference [%]
	Average Diameter [mm]	Coefficient of Variation [%]	Deviation from Nominal [%]	Average Diameter [mm]	Coefficient of Variation [%]	Deviation from Nominal [%]	
0	1.948	11.18	−2.58	1.798	9.45	−10.10	8.34
45	1.704	3.14	−14.79	1.768	5.78	−11.60	−3.62
90	1.688	3.04	−15.61	1.696	6.86	−15.20	−0.47

**Table 2:** Influence of the measurement direction.

Growth Direction [°]	Measurement Direction [°]	Average [mm]	Coefficient of Variation [%]	Deviation from Nominal [%]	Aspect Ratio
0	0	1.743	2.96	−12.85	1.24
	90	2.154	4.09	7.69	
45	0	1.714	2.78	−14.31	0.99
	90	1.695	3.37	−15.27	
90	0	1.666	2.69	−16.69	1.03
	90	1.709	2.83	−14.53	

## Data Availability

The data presented in this study are available on request from the corresponding author. The data are not publicly available due to privacy restrictions.
